# Testing VHF/GPS Collar Design and Safety in the Study of Free-Roaming Horses

**DOI:** 10.1371/journal.pone.0103189

**Published:** 2014-09-08

**Authors:** Gail H. Collins, Steven L. Petersen, Craig A. Carr, Leon Pielstick

**Affiliations:** 1 U. S. Fish and Wildlife Service, Sheldon-Hart Mountain National Wildlife Refuge Complex, Lakeview, Oregon, United States of America; 2 Department of Plant and Wildlife Services, Brigham Young University, Provo, Utah, United States of America; 3 Department of Animal and Range Sciences, Montana State University, Bozeman, Montana, United States of America; 4 Doctor of Veterinary Medicine, Burns, Oregon, United States of America; University of Tasmania, Australia

## Abstract

Effective and safe monitoring techniques are needed by U.S. land managers to understand free-roaming horse behavior and habitat use and to aid in making informed management decisions. Global positioning system (GPS) and very high frequency (VHF) radio collars can be used to provide high spatial and temporal resolution information for detecting free-roaming horse movement. GPS and VHF collars are a common tool used in wildlife management, but have rarely been used for free-roaming horse research and monitoring in the United States. The purpose of this study was to evaluate the design, safety, and detachment device on GPS/VHF collars used to collect free-roaming horse location and movement data. Between 2009 and 2010, 28 domestic and feral horses were marked with commercial and custom designed VHF/GPS collars. Individual horses were evaluated for damage caused by the collar placement, and following initial observations, collar design was modified to reduce the potential for injury. After collar modifications, which included the addition of collar length adjustments to both sides of the collar allowing for better alignment of collar and neck shapes, adding foam padding to the custom collars to replicate the commercial collar foam padding, and repositioning the detachment device to reduce wear along the jowl, we observed little to no evidence of collar wear on horses. Neither custom-built nor commercial collars caused injury to study horses, however, most of the custom-built collars failed to collect data. During the evaluation of collar detachment devices, we had an 89% success rate of collar devices detaching correctly. This study showed that free-roaming horses can be safely marked with GPS and/or VHF collars with minimal risk of injury, and that these collars can be a useful tool for monitoring horses without creating a risk to horse health and wellness.

## Introduction

Free-roaming horse (*Equus caballus*) management is a complex issue incorporating social, economic, emotional, political, and environmental factors. The complexity associated with developing appropriate free-roaming horse management practices has highlighted the need for an improved understanding of free-roaming horse behavior, habitat use, resource impacts, and movement patterns across the landscape. Global positioning system (GPS) collars offer a robust tool to track horse activity with high spatial and temporal resolution. In 1994, Lotek Engineering introduced the first animal-based GPS tracking device [1]. Since its initial development, GPS collar technology has been widely adopted and significantly improved through reduced collar and receiver size, lowered collar weight, increased longevity (battery life), lowered costs, and increased data storage and retrieval capacity [1].

Very high frequency radio (VHF) and GPS collar technologies are commonly used by wildlife managers and researchers to track and monitor a diversity of wildlife species, ranging from migratory birds [2] and small mammals to large herbivores and carnivores [3]. World-wide, over the past 30 years, thousands of individuals of a variety of species have been captured and collared to study ecological dynamics and behavior [4]. Examples of studies involving large numbers of collared ungulates include moose (*Alces alces*; [5]–[10]), elk (*Cervus canadensis*; [11]–[13], bison (*Bison bison*; [14]–[15], white-tailed deer (*Odocoileus virginianus*; [16], mule deer (*O. hemionus*; [17]–[18] and barren-ground caribou (*Rangifer tarandus*; [19]–[20]. Collars have also been used to track a variety of livestock species, in particular cattle (*Bos primigenius*; [21]–[25].

In contrast, few studies report the use of VHF or GPS collar technology in the study of wild *Equids*, however, those that have incorporated this technology have successfully generated data for the study of animal movement, behavior, and habitat use. For example, Siniff et al. [26] deployed 89 collars with and without VHF transmitters on free-roaming horses in western Nevada to monitor foaling and mortality rates. Kaczensky et al. [27] successfully deployed 16 VHF/GPS collars with pre-programmed timed-release detachment devices on nine Przewalksi's horses (*Equus ferus przewalskii*) and seven Asiatic wild asses (*Equus hemionus onager*) in the Mongolian Gobi. In the Australian outback, Hampson et al. [28]–[29] placed VHF/GPS collars on 12 feral horses to track travel patterns between feeding grounds and water holes. Eight collars were placed on adult female plains Zebras (*Equus burchelli antiquorum*) to test the influence of collar weight on animal behavior and location error [30].

Despite the technology's widespread application, in the United States collars have been barred from use on free-roaming horses that are under the jurisdiction of the Bureau of Land Management (BLM) because of previous incidents of collar-related injuries to horses fitted with VHF collars. The BLM is responsible for management of the majority of free-roaming horses in the United States. Problems included ill-fitting collars and, more importantly, a lack of collar removal that resulted in horses wearing collars for extended periods causing significant injury (personal communication Wild Horse and Burro Research Committee, 2010). Horse necks increase in width from the base of the head (lower mandible/axis) to the point of neck attachment above the shoulders and withers. Collars that are attached too low on the neck towards the withers have high potential for significant movement and associated injury. This is primarily due to slippage that occurs as collars slide up and down the neck while feeding, walking, and running. This rubbing motion can create hair wear and if excessive, open wounds and sores.

In the United States, free-roaming horse welfare and safety is a paramount management issue and because of safety concerns, there has been a limited use of VHF/GPS collar technology for improving our understanding of free-roaming horse ecology and management. The purpose of this study was to evaluate VHF/GPS collars and assess their potential to cause injury to free-roaming horses. This includes an assessment of collar design, degree of injury, and collar detachment reliability. Note that the terms free-roaming, free-ranging, feral, and wild horse are used interchangeable by horse management agencies and in scientific literature, however, these terms all refer to horses that live in an untamed state but have ancestors that were once domesticated. In this paper, we refer to these animals as free-roaming horses.

## Methods

### Study area

**Figure 1:**
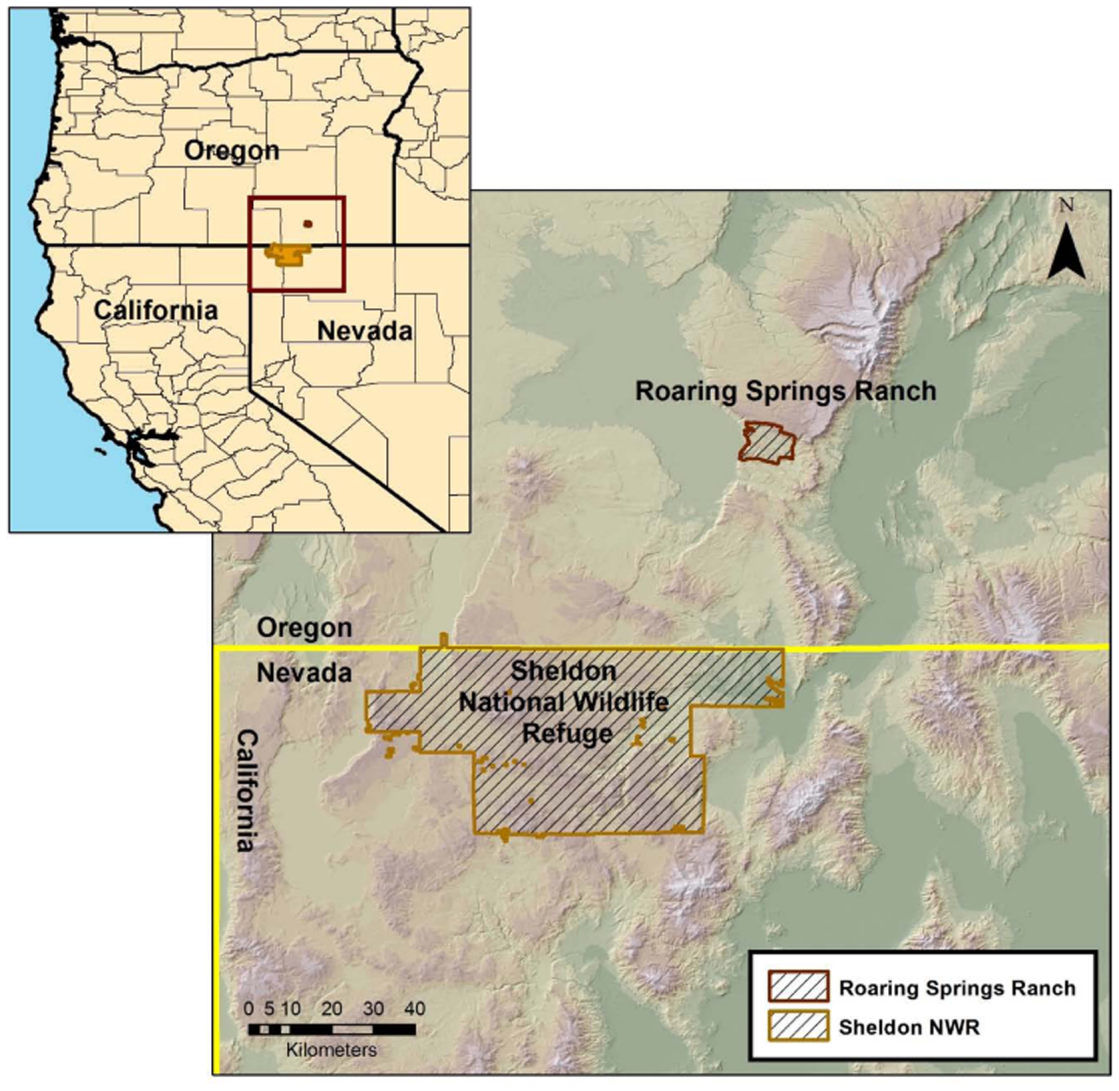
Pilot and in-field study site locations. The Roaring Springs Ranch (pilot study) is located in southeast Oregon. The Sheldon National Wildlife Refuge (in-field study) is located in northwestern Nevada.

We used two separate study locations to evaluate the utility and safety of VHF/GPS collars in characterizing free-roaming horse movement and habitat use patterns: the Roaring Springs Ranch (RSR) in southeast Oregon and the Sheldon National Wildlife Refuge (SNWR) in northwest Nevada (Figure 1). The RSR served as our initial pilot study, testing collar design on domestic, free-roaming horses while the SNWR provided an opportunity to evaluate collars on feral, free-roaming horses.

#### Pilot Study – Roaring Springs Ranch

The RSR is located on the western side of Steens Mountain (42.36108° N 118.78923° W) in the northernmost extent of the Great Basin, High Desert Ecological Province [31] (Figure 1). The privately owned ranch covers approximately 171,991 hectares, ranging in elevation between 1,386–2,324 m. The area receives approximately 32 cm of precipitation annually, primarily during winter and early spring months. The sagebrush-steppe vegetation is dominated by mountain big sagebrush (*Artemisia tridentata* Nutt. *ssp*. *vaseyana* (Rydb.) Beetle), bottlebrush squirreltail (*Elymus elymoides* (Raf.) Swezey), Idaho fescue (*Festuca idahoensis* Elmer), bluebunch wheatgrass (*Pseudoroegneria spicata* (Pursh) A. Love), and Sandberg's bluegrass (*Poa secunda* J. Presl).

#### In-Field Study – Sheldon National Wildlife Refuge

The SNWR is located along the southern Oregon border to the north and near the California border to the west (41.72079°N latitude, 119.39703°W). The SNWR covers approximately 232,694 hectares of sagebrush-steppe habitat with elevations that range between 1,200–2,100 m. Average annual precipitation ranges between 18 and 33 cm. Plant communities in this region are dominated by big sagebrush (*A. tridentata* ssp. *vaseyana* and ssp. *wyomingensis* Beetle and Young), little sagebrush (*A. arbuscula* Nutt.), antelope bitterbrush (*Purshia tridentata* (Pursh) DC.), bottlebrush squirreltail, Idaho fescue, and bluebunch wheatgrass. Several tree species such as quaking aspen (*Populus tremuloides* Michx), western juniper (*Juniperus occidentalis* Hook.), and curl-leaf mountain mahogany (*Cercocarpus ledifolius* Nutt.) are found in scattered stands throughout the SNWR.

### Field data collection

#### Pilot study – Roaring Springs Ranch

**Figure 2:**
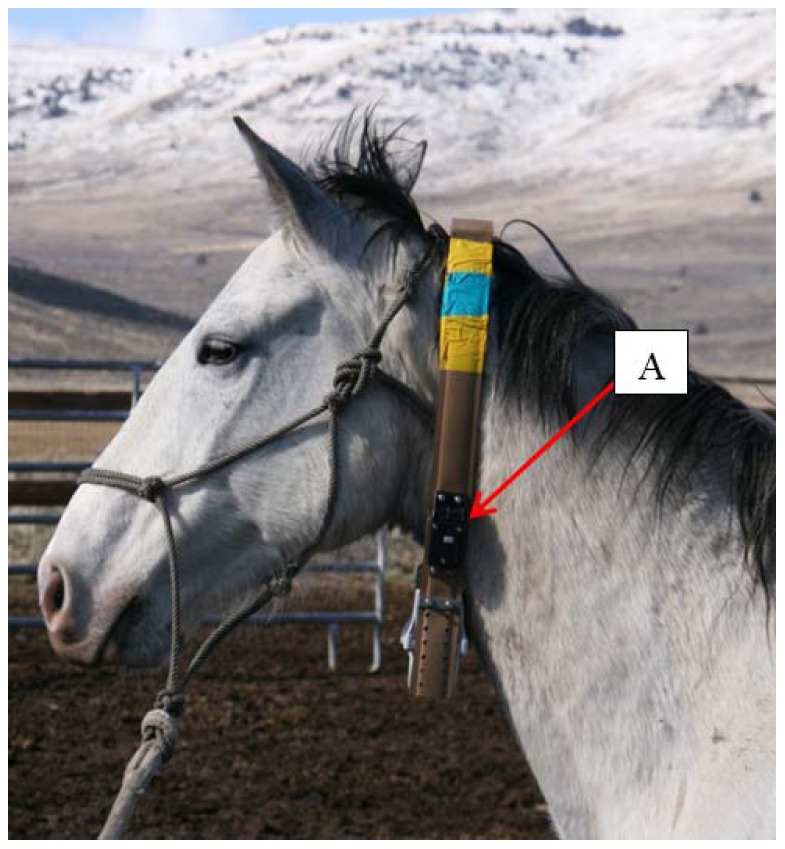
An adult domestic horse fitted with a commercially available VHF/GPS collar. A: location of remote-detonated detachment device immediately behind the jowl and jugular furrow prior to modification.

**Figure 3:**
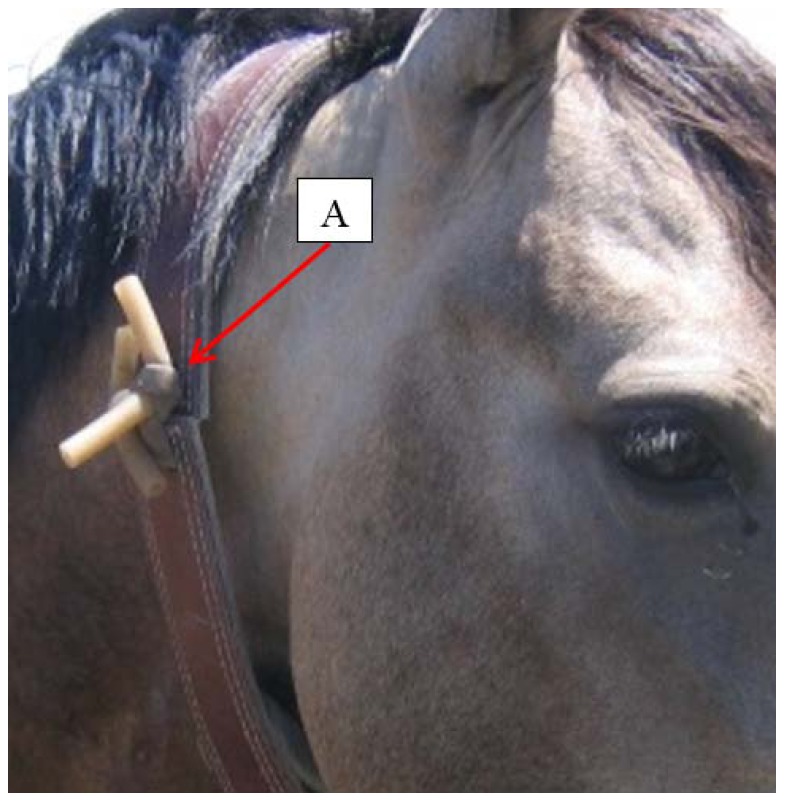
An adult domestic horse fitted with a custom-built VHF/GPS collar. A: location and design of breakaway detachment device constructed of two pieces of parallel rubber tubing.

Between March and August 2009, we deployed six commercial (Lotek GPS3300; Figure 2) and 11 custom-built (Figure 3) VHF/GPS collars on 12 privately-owned domestic horses. Five individuals were collared twice during this portion of our study (Table 1). Two weeks after the initial collar deployment in March 2009, six custom collars were removed in order to recover the data. A second deployment of five custom collars which were constructed with breakaway detachment devices occurred in June 2009 (Table 1). Six horses were mares and 6 were geldings, ranging in age from 6–17 years old. Two of the mares also had foals in attendance. The design and construction of the custom-built collars followed Clark et al. [32] and were used because they were more cost effective than commercial collars and because this design has experienced increased use in the field. These horses were allowed to range freely as a group within a 12,140 hectare fenced pasture, and rounded-up by ranch personnel every two to four weeks in order to evaluate each horse for potential injuries as a result of the collars. For the purposes of this study, these animals were assumed to provide a fair representation of free-roaming horses, including the presence of a dominant male horse, while the ability to regularly and safely handle these individuals afforded us the opportunity to evaluate the potential for injury associated with collar placement and fit.

**Table 1:** Types and dates of VHF/GPS collars deployed on privately-owned domestic horses March-August 2009, Roaring Springs Ranch, OR.

Horse ID	Commercial (with breakaway^1^)		Custom-built (no breakaway)		Custom-built (with breakaway^2^)	
	Deployed	Retrieved	Deployed	Retrieved	Deployed	Retrieved
Mares						
Cabana	26-Mar-09	29-Jun-09				
Docs	26-Mar-09	29-Jun-09			29-Jun-09	10-Aug-09
Erica			26-Mar-09	09-Apr-09	29-Jun-09	10-Aug-09
Jill	26-Mar-09	29-Jun-09			29-Jun-09	17-Aug-09
Lil' Bailey			26-Mar-09	09-Apr-09	29-Jun-09	17-Aug-09
Shirley			26-Mar-09	09-Apr-09	29-Jun-09	17-Aug-09
Geldings						
Club			26-Mar-09	09-Apr-09		
Gus			26-Mar-09	09-Apr-09		
Scorpio	26-Mar-09	29-Jun-09				
Tickers	26-Mar-09	29-Jun-09				
Wade	26-Mar-09	21-Apr-09				
Watermelon	21-Apr-09	29-Jun-09	26-Mar-09	09-Apr-09		

1 Remotely-detonated breakaway detachment device.

2 Breakaway detachment device constructed of rubber tubing.

Collar detachment or drop-off devices are designed to break open the collar band and allow it to fall free from the animal's neck without direct human contact. Proper deployment of the detachment device prevents the need to recapture and further handle the animal. Two of the most common types of collar detachment devices are either a remotely-detonated device which uses a remote trigger to activate a small explosive to separate the collar band or a remote timed-release device which mechanically separates the collar band within a pre-programmed time period.

Occasionally these types of collar detachment devices fail to function correctly, thus a common alternative is to use breakaway or rot-away detachment devices such as rubber tubing or leather or cotton spacers. As the material is exposed to the sun and other environmental elements, it becomes brittle and breaks apart allowing the collar to fall from the animal's neck. For this study, we selected to use a breakaway device constructed of ½ cm diameter rubber tubing. The collar band was cut in half and two holes drilled into each cut end. Two pieces of tubing were then threaded in parallel through each pair of holes and tied into a knot to close the cut collar band ends (Figure 3).

During the pilot study, we tested the efficacy of two types of collar detachment devices. Five of the custom-built collars were outfitted with a breakaway collar detachment device constructed of rubber tubing (Figure 3) and all six of the commercial collars were outfitted with a commercial remotely-detonated device (Figure 2). The original six custom collars did not have detachment devices (Table 1).

#### In-field study – Sheldon National Wildlife Refuge

Using modifications to the collar design developed during the pilot study we attached six commercial (Lotek GPS3300) and eight custom collars VHF/GPS collars to free-roaming horses within the Sheldon National Wildlife Refuge in August 2009 (Table 2). Horses were gathered as a group and herded into corrals via helicopter and fourteen adult mares were collared and released at the site of capture. All collared mares were also implanted with a unique micro-chip prior to release for future identification. In October 2010, horses were again gathered by helicopter. We attached commercial collars (Lotek GPS3300) to six adult mares, four of which were collared previously in August 2009, and all were released at the site of capture (Table 2).

**Table 2:** Type of collar, type of detachment device, and date dropped for VHF/GPS collars deployed on free-roaming horses in 2009 and 2010, Sheldon National Wildlife Refuge, NV.

	August 2009 Deployment			October 2010 Deployment		
Horse ID	Collar Type	Activated detachment device	Date Dropped	Collar Type	Activated detachment device	Date dropped
192	Custom	Timer	Sept 2010			
206	Custom	Timer	Sept 2010			
300	Custom	Timer/Breakaway^1^	<Sept 2012			
242	Custom	Timer/Breakaway^1^	<Sept 2012			
320	Custom	Timer/Breakaway^1^	<Sept 2012			
255	Custom	Timer/Breakaway^1^	<Sept 2012			
280	Custom	Timer/Breakaway^1^	<Sept 2012			
R217	Custom	Timer/Breakaway^1^	<Oct 2010	Commercial	Timer	Failed^2^
004	Commercial	Breakaway	Jun 2010			
055	Commercial	Breakaway	May 2010			
019	Commercial	Breakaway	Mar 2010			
R067	Commercial	Breakaway	Mar 2010	Commercial	Timer	Oct 2012
R028	Commercial	Breakaway	Dec 2009	Commercial	Timer	Nov 2011
R043	Commercial	Breakaway	Mar 2010	Commercial	Timer	Nov 2011
N004				Commercial	Timer	Failed^2^
N054				Commercial	Timer	Oct 2012

1 Individual captured at a later date without the collar, it is unknown which detachment device activated first.

2 Timed-release detachment devices failed, collars were manually removed during subsequent captures.

**Figure 4:**
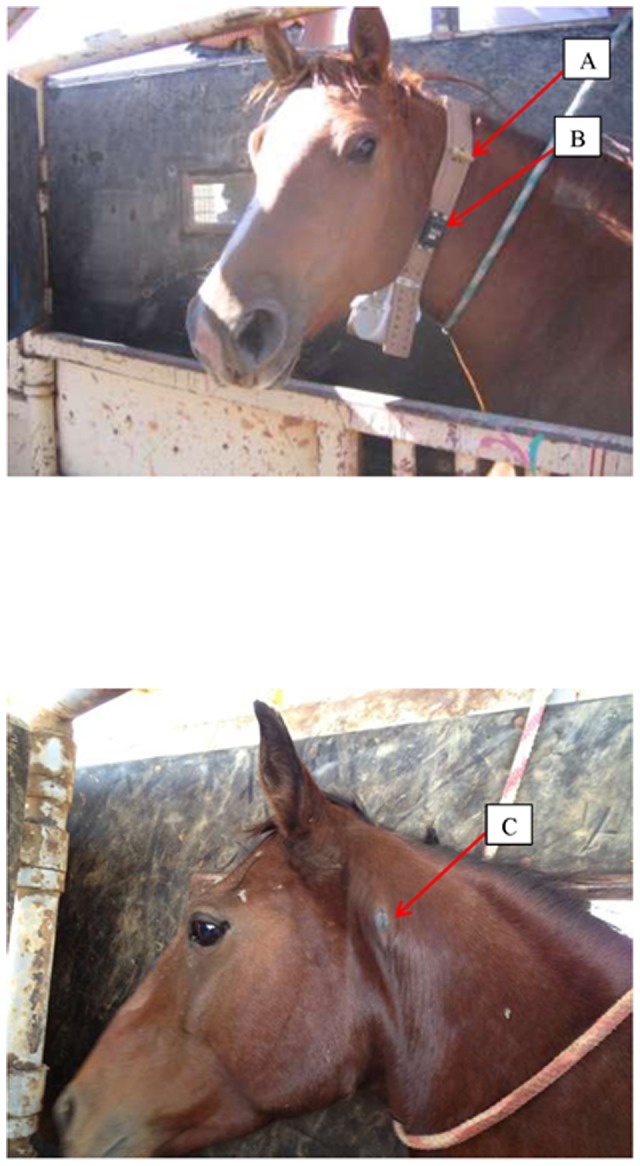
An adult free-roaming horse during collar deployment and a second horse after collar detachment. First photo: area to right of rope is where a micro-chip was implanted, A: breakaway device constructed of rubber tubing, B: breakaway device using pre-programmed timed-release; Second photo: C: only a small callus and typical slight hair wear is in evidence along the upper portion of the neck and mane where the collar was situated for 3 years.

All collars were deployed on adult mares ranging in age from four to 24 years old and ten of the mares were attended by foals at the time of collaring. During the initial collar deployment in August 2009, all collars were outfitted with both a breakaway device (i.e., rubber tubing, ½ cm diameter) and a commercially available remote timed-release device (Lotek mini drop off) set to detach with a delay of approximately 53 weeks (Figure 4).

Following the initial deployment in August 2009, all of the breakaway devices installed on the six commercial collars activated prior to the pre-programmed date for the timed-release devices. The commercial collars were retrieved from the field, and were refurbished by the manufacturer for re-deployment in October 2010 on SNWR. Because the timed-release devices were still intact and operating when retrieved, they were not replaced during refurbishment and instead reset in the field according to the manufacturer's instructions. The refurbished commercial collars deployed in October 2010 were outfitted with only the reset timed-release detachment devices.

### Ethics statement

This study was carried out in strict accordance with the protocol approved by the Brigham Young University Institutional Animal Care and Use Committee (USDA Permit Number: 27-2956, BYU Protocol number 08-081). No permits were required to perform this research. The pilot study was performed on private land under the authority of the landowner while the field study was performed with permission and guidance of the USFWS on the Sheldon National Wildlife Refuge. Our research did not involve Federally- or State-listed endangered species or species of concern. The owners of the domestic horses granted permission for their animals to be use in this study.

## Results

### Pilot study – Roaring Springs Ranch

Hair wear includes thinning and breakage of hair where friction is enough to provide visible evidence of contact with the collar. In approximately 30% of the cases, hair wear at the RSR pilot study was sufficient enough to expose the skin, however, as this took place over an extended period, the underlying skin adapted by thickening and strengthening the epidermal structure and the skin remained pliable with no evidence of callus formation or loss of elasticity. In no case was the skin integrity compromised. Collars were placed high on the neck to reduce collar movement while also limiting the potential for collar damage associated with horse to horse interactions (e.g., kicking), or getting the collar caught in vegetation or on fences. Moreover, collaring only mature animals prevented the possibility of an immature animal outgrowing the collar and causing injury. During the pilot study, one of our main goals was to evaluate study horses for potential issues or injury attributed to the collar. The collars were installed behind the head and ears (Figure 2) and initially adjusted in tightness to approximately 2.5-5 cm spacing between the neck and the collar.

Observations over the first 4 weeks following deployment included: 1) slight to moderate abrasions or callus on the jowl at the jugular furrow; and 2) slight to moderate wearing in the hair at the widest point of the neck and on the mane. We also found that the remote collar detachment device was causing skin irritation by rubbing along the jowl (Figure 2). As a result, we made the following modifications to the collar design: 1) to reduce swinging, side-to-side collar movement, the collar shape was narrowed by adding collar length adjustments to both sides of the collar which created a more natural oval shape (as opposed to round or off-centered) in alignment with the shape of a horse's neck; 2) to augment the oval shape in the custom collars we added foam padding to the upper portion of the VHF/GPS box; 3) repositioning the remote collar detachment device higher on the neck to avoid contact with the jowl; and 4) tightening the collar to 1.3 cm spacing on a flexed neck to reduce collar movement up and down along the neck. Following these modifications we observed only slight wear in the hair along the neck and mane of the horses with no abrasions or calluses even after several months of wear. It is important to note that all of the domestic horses gained substantial weight during the course of the study owing to naturally improving range conditions through the spring and summer, and there were no associated issues with the tightness of the collar.

During the pilot study, two of the breakaway devices detached before 40 days while the remaining three collars with breakaway detachments were intact when the study ended and the collars removed in August 2009. Six of the seven (87%) remote-detonated collar detachment devices detached as expected after 93 days deployed. The reason for the single failure is unknown.

### In-field study – Sheldon National Wildlife Refuge

During the pilot study, we documented that several of the domestic geldings were able to slip the upper portion of the collar band over one or both ears; we did not see this occur with any of the mares. The exact reason for this is unknown, but to reduce the potential for harm to free-roaming male horses, the decision was made to only collar mares during the in-field portion of the study. In addition, we suspected that intrasexual fighting among male horses would likely result in substantial collar damage [29].

For the custom-built collars deployed in August 2009, the VHF malfunctioned almost immediately and stopped transmitting from seven of the eight collars making the collars irretrievable from the field. The remaining collar was successfully retrieved field via a functioning VHF transmitter and a second collar was found by chance and recovered by a member of the public. All eight horses who received custom-built collars were recaptured during subsequent helicopter gathers between 2010 and 2012. None of these individuals were wearing a collar when recaptured and under examination showed no evidence of lasting injury as a result of the collar (e.g., calluses, scars). Because the collars were irretrievable from the field due to the VHF malfunction, it is unknown exactly when and which collar detachment device activated first (the breakaway or timed-release), but the end result was the same with all collars detaching successfully.

The VHF transmitters in the commercial collars transmitted a signal throughout the study. For the commercial collars deployed in August 2009, all six had their breakaway device detach before the set remote timed-release date of approximately 370 days since deployment, ranging from 122 to 254 days (Table 2). The commercial collars were then refurbished by the manufacturer and redeployed with only the field-reset timed-release detachment device in October 2010. For those redeployments, four of the six remote timed-release devices functioned properly although at roughly two different time intervals (386 days and 703–717 days; Table 2). The remaining two remote timed-release devices failed to activate altogether.

Collar wear during the in-field study was as expected from that observed following the collar modifications during the pilot study. During the course of the in-field study, we located the bands with marked individuals via VHF by air or ground every 1 to 2 months, and all of the mares appeared from a distance to be in good health and wearing the collars without issue. These observations included individuals wearing collars that had stopped transmitting a VHF signal, but were able to be located due to their association with mares whose collars were transmitting correctly. Fourteen mares were incidentally captured during subsequent gather efforts and under direct examination showed very little to no evidence of effects related to the collar regardless of type of collar deployed. If evidence of the collar was present, we observed only a small callus or a slight wearing of the hair along the neck and mane. Of the two individuals whose timed-release detachment device did not function properly, one was recaptured in 2013 still wearing the collar and showed no injury or damage as a result of the collar even after 3 years of continuous wear (Figure 4).

## Discussion

Ill-fitting collars and problems associated with them clearly influence research results and have implications for ethics within the wildlife profession [4]. With that in mind, we set out to test the efficacy and safety of collar deployment on a high profile species, the free-roaming horse in the United States. This work demonstrated that free-roaming horses can be safely collared using an appropriately modified design with minimal to no detectable physical impact to the horse.

We tested the custom-built collars due to the greater cost savings however, we experienced issues with their functionality, likely due to inexperience with the construction and potentially rougher handling by horses than other species. We observed several instances where a commercial collar took a direct kick from another horse and continued to function normally. For those custom-built collars we could recover, in several locations soldered joints in the electronics were broken leading us to suspect there were flaws in the construction. Collars built following the Clark et al. [32] design are effective in monitoring animal movement [33], however based on our experiences, these collars should be constructed by experienced technicians.

In evaluating the collar detachment devices, the collars detached successfully in 89% of the cases. We suspect issues related to failure in the second deployment of the timed-release devices were related to our attempts to field-reset them, where two detached at the expected time, two detached at twice the expected time, and two failed to detach. For those collars that were not removed by either a remote-detonated or a timed-release device, the fail-safe breakaway devices were 100% effective. The downside of the breakaway devices is a lack of control over when the collar will detach, which in our environment ranged roughly between 4 and 8 months. This timing would be expected to be variable depending on the thickness of the rubber tubing used, climate (temperature, sun intensity) of the study area, and time of year (winter vs. summer). We expect that thicker tubing, would last longer, however, tubing thicker than the diameter tested in this study would be difficult to tie into the collar and may subsequently falter.

The potential exists for horses to be injured when collars get caught in trees or fencing. Although we did not specifically monitor for these events, we saw no evidence of any injuries that could be attributed to horses being trapped in fencing or trees because of collars. Moreover, breakaway devices may provide protection against this type of injury, however more research specifically on the strength of the rubber tubing is required to fully address this question.

Based on our experience in this study, collar fit is an important consideration when using VHF/GPS collar on free-roaming horses. By maintaining a natural oval shape and fitting the collar high on the neck, collar movement and subsequent wear was significantly reduced. In addition, collar detachment devices should be installed along the collar in locations to ensure they do not cause excessive wear along the horse's jowl.

Injury to study animals can occur when using GPS/VHF collars. Krausman et al. [4] observed collar related injuries on wild sheep and mule deer and indicated that, as in our study, injuries were related to ill-fitting collars. Once collars were properly aligned with the shape of the neck and protruding fasteners made flush with the collar straps, injury rates were substantially reduced, however wild sheep were apparently more susceptible to collar wear injury [4]. Moreover, many collar related injuries appear associated with changing neck size, either from juvenile growth or physiological changes including periods of hibernation [34] or breeding [35]. Designs with expandable collar straps or break-away devices have been proposed to address neck size changes [34]–[35]. For horses, there is a risk of collar-related injury associated with increased neck size when juveniles are collared or if neck size expands as body condition improves with high quality and abundant spring and summer forage. In our case, we chose not to collar juveniles and saw no evidence of collar related injuries associated with improved body condition. However, break-away devices should be considered on collars as a way to prevent potential neck size issues, particularly if collars are being deployed on juvenile animals. In most cases, it does appear that with properly fitted collars, GPS/VHF collars can be effectively and safely used in free ranging animal studies without significant collar related injury to study animals. In our review of collar-based wild *Equid* studies, none reported observations of significant collar-related injuries to study animals [26]–[27], [30], [36] and both Brooks et al. [30] and Gerard et al. [36] indicated that there was no evidence negative impacts associated with collars on either zebras or feral horses.

Although not reported in this paper, the data collected by our GPS collars provided useful information with respect to the effectiveness of geospatial data in evaluating free-roaming horse ecology including landscape-level movement patterns [37] and habitat selection [38]. These results, along with other GPS collar based studies of horse behavior in Canada [36] and Australia [28] and other wide-ranging ungulates [11]–[13], [19]–[20], demonstrate the effectiveness and utility of the data generated by collars in the study of wild and free-roaming horses.

### Implications

The use of VHF/GPS collar technology is critical to understanding how free-roaming horses, particularly in the Western United States, move across the larger landscape and use increasingly scarce resources. Lack of this information has contributed to the management complexity of this species [39]. Applying this technology to the study of free-roaming horses will provide the opportunity to better understand horse resource use, habitat preference, home range, and movement patterns and can be incorporated into investigations of social structure and herd or band dynamics as well as behavioral modifications associated with reproductive management including contraceptive use and sterilization. Moreover, an improved understanding of horse ecology is critical to the appropriate management of natural resources in the western US and is needed to address questions related to predator relationships, competition for habitat, and interactions among and between horses, wildlife, and livestock. Our study indicates that VHF/GPS collar technology can be safely used as a tool in the development the body of knowledge required to effectively manage wild and free-roaming horses.
